# Risk perception and use of personal care products by race and ethnicity among a diverse population

**DOI:** 10.14324/111.444/ucloe.3038

**Published:** 2024-05-13

**Authors:** Julia Mandeville, Zeina Alkhalaf, Charlotte Joannidis, Michelle Ryan, Devon Nelson, Lesliam Quiros-Alcala, Matthew O. Gribble, Anna Z. Pollack

**Affiliations:** 1 Department of Global and Community Health, College of Public Health, George Mason University, Fairfax, VA, USA; 2 Department of Environmental Health and Engineering, Johns Hopkins University, Baltimore, MD, USA; 3 Division of Occupational, Environmental & Climate Medicine, University of California, San Francisco, CA, USA

**Keywords:** personal care products, diversity, perception of safety, risk perception, use frequency, race and ethnicity, people and their environment, health

## Abstract

Personal care products can contain phthalates, parabens and other endocrine-disrupting chemicals. However, information on perception of risks from personal care product use and how use varies by race and ethnicity is limited. We evaluated differences in personal care product use and risk perception in a diverse sample of participants recruited from a US college campus and online. A self-administered questionnaire captured information on sociodemographic factors, personal care product use trends and perception of risk associated with them. Pearson’s chi-square and Fisher’s exact tests were used to determine differences in personal care product use and risk perception by race and ethnicity. Ordered logistic regressions were performed to measure associations between personal care product use frequency across racial/ethnic categories. Participant (n = 770) mean age was 22.8 years [standard deviation ± 6.0]. Daily use of make-up (eye = 29.3%; other = 38.0%; all = 33.7%) and skincare products (55%) was most frequently reported among Middle Eastern and North African participants. Non-Hispanic Black participants reported the highest daily use of hairstyling products (52%) and lotion (78%). Daily make-up use was more frequently reported among females (41%) than males (24.6%). Levels of agreement were similar across racial and ethnic groups, that personal care product manufacturers should be required to list all ingredients (≥87%). There were significant associations between the frequency of use of some personal care products and racial/ethnic categories when the use frequencies of participants from other racial/ethnic categories were compared to the use frequency of non-Hispanic White participants. There were significant differences in daily use frequency, levels of trust, perception of safety and health risks associated with personal care products by race and ethnicity, underscoring that there may be different sources of exposure to chemicals in personal care products by race and ethnicity.

## Key findings

1. Daily use frequency of make-up and skin care products varied across racial and ethnic groups.
2. Perception of health risks associated with personal care product (PCP) use was less frequently reported by non-Hispanic White (NHW) participants compared to participants of other racial and ethnic groups.
3. Participants across the racial and ethnic group shared the sentiment that PCP manufacturers should be required to list all ingredients present.
4. Based on differences in use frequency and risk perceptions, there may be different sources of exposure to PCPs by race and ethnicity.
5. Further research is warranted to determine if these differences in use and risk perception between racial and ethnic groups are consistent.

## Introduction

Personal care products (PCPs) describe externally applied products that are typically used for cosmetic and/or hygienic purposes and are extensively used by consumers [1–3]. PCPs are regulated by the Food and Drug Administration (FDA) [1], but loopholes in ingredient labelling requirements allowed manufacturers to omit listing all ingredient PCP components [4]. This lack of ingredient labelling obscures consumers’ ability to know the contents of the products they use. Some chemicals found in PCPs include parabens, phthalates and environmental phenols [4–6], which can disrupt endocrine function, particularly raising concerns about their impact on reproductive system function and women’s health [7,8]. For example, parabens, used as antimicrobial ingredients in PCPs, have been detected in breast cancer tumours [9]. Furthermore, consumers may be unaware of many of these chemicals’ risks.

Continuous exposure to chemicals in these products and the lack of access to information about the chemicals included in PCPs and their dangers may pose undetermined substantial risks to consumers. Additionally, these risks have been found to differ by race and ethnicity [10]. Differences in PCP use across racial/ethnic groups may contribute to the cascading ill effects of health inequities and disparities, as people seek to conform to standards of idealised Whiteness – spending more money on and using more (in type and volume) PCPs to meet these socially imposed standards [11–13]. For example, non-Hispanic Black (NHB) women purchase nine times more ethnic hair and beauty products, including hair relaxers and straighteners, than other racial/ethnic groups and studies report higher use of hair products that contain endocrine disrupting compounds (EDCs) among Black women compared to non-Hispanic White (NHW) women [3,5,6,14]. Moreover, urinary biomarker concentrations of chemicals commonly found in hair and beauty products are also reported to be higher in NHB women compared to NHW women [3,15–19]. These findings pose significant public health concerns as exposures to EDCs may have considerable adverse health impacts, such as earlier menarche, breast development and pubic hair development, which may be linked with an increased risk of developing breast or endometrial cancer later in life [7].

Most research examining PCP-use patterns and associated exposures to endocrine disruptors among racial/ethnic groups has primarily focused on the comparison of African-American women to NHW women [20]. However, the inclusion of other racial/ethnic groups is essential as other studies indicate rapidly expanding use of and spending on PCPs among Latinos and Asian Americans, with the latter spending more on skin care products compared to NHW populations [10]. Moreover, information on consumer perception of risks from PCP use is limited.

Capturing information on risk perceptions around PCPs provides an understanding of how people make decisions in the purchasing and use of these items. Particularly as PCP labels may not necessarily list all ingredients, purchasing and use of products typically result from individual self-assessment of the product and assumptions guided by secondary information through social networks and cultural norms and practices [13,21]. This secondary information can be prone to misrepresentation and misinterpretation in addition to unintentional health-related consequences for users such as allergic responses [22,23], cancer [9,24,25] and infection risks [26–29]. In the present study, we aimed to examine PCP use and estimate differences in risk perception across racial/ethnic groups among a diverse population of United Staes (US) adults at a university.

## Methods

### Study participants

A total of 770 participants were recruited from the George Mason University, Fairfax, Virginia, campus in person and online. The survey was administered in 2013 and in 2016–2017. The George Mason University Institutional Review Board approved the study as exempt. Individuals who were 18 years or older were eligible to participate.

### Data collection

Data on demographics, PCP use and risk perception of PCPs were collected using a self-administered questionnaire. In-person recruitment took place on George Mason University’s Fairfax, Virginia, campus in 2013 and online recruitment in 2016–2017.

### PCPs use and risk-related information

The use of 23 individual PCPs was assessed (Appendix A). These were categorised into: (1) eye make-up, (2) other make-up (e.g., make-up primer, lip pencil, blush), (3) skin care (e.g., facial moisturiser, hand lotion, sunscreen), (4) hair products (e.g., hairstyling products), (5) manicuring (e.g., nail polish) and (6) fragrances (e.g., fragranced shampoo, fragranced shaving cream). Self-reported frequency of PCP use was collected using the following options: >1/day, 1/day, every other day, 2 times/week, 1/week, never or very rarely. Frequency of use categories were further coded as frequent (more than once a day or daily), moderate (every other day and twice a week) and infrequent (once a week, never or very rarely).

### Risk perception

Participants were provided with 18 statements to capture risk perception. These were categorised as follows: Regulation and protection (statements 1–4), Risk and safety (statements 5–8), Responsibility (statements 9–11), Trust (statements 12–16) and Transparency (statements 17–18) using a 5-point Likert scale to determine their perception of risk associated with PCP use (Appendix B). Responses were coded as agree (strongly agree and tend to agree), disagree (strongly disagree and tend to disagree) and unsure.

### Statistical analysis

We first summarised the demographic characteristics of study participants. Pearson’s chi-square and Fisher’s exact tests were used to determine if individual PCP use and perception of PCP safety varied by race and ethnicity, sex reported by participant and country of birth (Appendix C). To ascertain proportional odds of frequency of PCP use and racial/ethnic category, ordered logistic regression models of were run in R. Unadjusted models were run to assess the relationship between frequency of individual PCP use (never, weekly, daily) and race/ethnicity.

We repeated these models, controlling for age, country of birth, level of education and sex. To preserve power, the variable for country of birth was dichotomised as US vs. non-US born. The referent racial/ethnic category [odds ratio (OR) = 1] was the NHW group and statistical significance was defined as *p* < 0.05. Statistical analyses were performed with SAS statistical software (SAS 9.3, Cary, NC, USA) and in R Studio (Build 421 Posit Software, PBC, Boston, MA, USA).

## Results

### Participant characteristics

The mean age of participants in the sample was 22.8 years standard deviation (SD) ± 6.0 years (Table 1). Participants were diverse and comprised NHW (34.8%), Asian or Asian American (20.1%), non-Hispanic Black or African American (NHB) (14.2%), Latino (12.5%), Middle Eastern and North African (MENA) (6.5%), Multiracial (4.6%) and other (7.4%). The majority (65%) of participants identified as women, 33.5% as male and 1% as nonbinary or preferred not to answer. Just over one-third (35.4%) were born outside of the US, and 81.4% had a college education.

**Table 1.** Socio-demographic characteristics of survey participants (n = 770)^a^

Personal information	Mean ± SD
**Age (years)**	22.82 ± 6.03
	N (%)
**Ethnicity/Race**	n = 768
MENA	50 (6.5)
Asian or Asian American	154 (20.1)
Black or African American	109 (14.2)
Hispanic or Latino	96 (12.5)
Multiracial	35 (4.6)
NHW or Caucasian	267 (34.8) 57 (7.4)
Other	
**Gender**	n = 769
Female	502 (65.3)
Male	258 (33.5)
Nonbinary/Prefer not to answer	9 (1.2)
**Country of birth**	n = *767*
US	525 (68.4)
Outside of US	242 (31.6)
**Education**	n = 769
High school/some college	626 (81.4)
College graduate	117 (15.2)
Other	26 (3.4)

^a^Participants were enrolled in two phases: 2013 and 2016–2017.

### Daily PCP use by race and ethnicity

Average daily use of all included PCP preparations by category ranged from 39.3% for Asian participants to 48.4% for MENA (Table 2). There was a statistically significant difference in the use of 14 individual PCPs [(1) brow pencil, (2) lip balm/lipstick/lip gloss, (3) brush/bronzing make-up, (4) lip pencil, (5) make-up remover, (6) hand/body lotion, (7) sunscreen, (8) general hairstyling products, (9) deodorant/antiperspirant, (10) fragranced shampoo, (11) fragranced conditioner, (12) fragranced facial soap/cleanser, (13) perfume/cologne/body spray, (14) fragranced hand soap] by racial and ethnic group. On average, the products with the most frequent daily use across the sample were deodorant or antiperspirant (81.4%), fragranced hand soap (72.3%) and fragranced soap or body wash (66.1%). Products with the lowest daily use reported included lip pencil (9.7%), make-up primer (14.3%) and eye shadow (15%) (Table 2).

**Table 2.** Daily use frequency of PCPs by race and ethnicity reported by surveyed participants from 2013 and between 2016 and 2017^a^

PCP preparation category	Individual PCP	MENA n = 50 (%)	Asian or Asian American n = 154 (%)	Black or African American n = 109 (%)	Hispanic or Latino n = 96 (%)	Multiracial n = 35 (%)	NHW n = 267 (%)	Other n = 57 (%)	*p*-value
Eye make-up	Eyeliner (liquid or pencil)	42.0	33.0	27.0	33	32.0	29.0	30.0	0.64*
	Brow pencil	29.0	20.0	19.0	18	21.0	13	14.0	0.02**
	Eye shadow	17.0	15.0	14.0	14.0	15	21	12	0.53*
	**Average eye make-up use**	**29.3**	**22.7**	**20.0**	**14.0**	**26.5**	**29.0**	**22.0**	
Other make-up	Make-up primer	19	14	9	16	21	12	9	0.27**
	Liquid foundation or concealer	36	27	16	22	35	30	20	0.07*
	Powder foundation or concealer	31	25	17	24	24	24	16	0.53*
	Lip balm, lipstick or lip gloss	69	63	70	55	71	57	50	0.01*
	Blush or bronzing make-up	50	29	25	32	35	27	29	0.05*
	Lip pencil	17	10	10	9	12	5	5	0.01**
	Make-up remover	44	30	18	33	26	25	21	0.04*
	**Average other make-up use**	**38.0**	**28.3**	**23.6**	**27.3**	**32.0**	**25.7**	**21.4**	
All make-up	**Average make-up use**	**35.4**	**26.6**	**22.5**	**25.6**	**29.2**	**24.3**	**20.6**	
Manicuring	**Nail polish^b^**	**66**	**72**	**61**	**57**	**59**	**66**	**77**	0.13**
Body care: skin care	Facial moisturiser	62.0	63.0	51.0	52.0	50.0	49.0	58.0	0.06*
	Hand or body lotion	67.0	63.0	78.0	60.0	59.0	46.0	61.0	<0.001*
	Sunscreen	36.0	26.0	15.0	24.0	18.0	22.0	16.0	<0.001*
	**Average skin care product use**	**55.0**	**50.7**	**48.0**	**45.3**	**42.3**	**39.0**	**45.0**	
Hair	General hairstyling products	**34**	**30**	**52**	**32**	**26**	**28**	**42**	<0.001*
Other body care	Deodorant or antiperspirant	85	60	86	87	85	90	77	<0.001**
Fragrance	Fragranced shampoo	40	46	21	45	44	53	45	<0.001**
	Fragranced conditioner	35	37	17	41	42	43	39	<0.001*
	Fragranced soap or body wash	69	61	69	65	74	67	58	0.55**
	Fragranced facial soap or cleanser	52	56	50	55	59	41	52	0.005*
	Fragranced shaving cream	15	19	16	20	29	12	16	0.09*
	Perfume or cologne or body spray	77	46	57	56	53	36	43	<0.001*
	Fragranced hand soap	87	64	68	74	85	73	55	<0.001**
	**Average fragranced product use**	**53.6**	**47.0**	**42.6**	**50.9**	**55.1**	**46.4**	**44.0**	
	**Average PCP use by race and ethnicity**	**46.9**	**39.5**	**37.7**	**40.2**	**42.4**	**37.8**	**36.7**	

^a^Participants who responded either ‘more than once a day’ or ‘daily’ use were grouped together to comprise daily use.

^b^Comparing ‘never’ use to all other categories.

*p-value was calculated using a chi-squared test.

**p-value was calculated using Fisher’s Exact test.

Bold numbers indicate the average use per personal care product (PCP) preparation category. Bold and italic numbers indicate statistical significance.

### Daily PCP use by self-reported sex-assigned-at-birth

There were also statistically significant differences between the frequency of use of PCPs and sex reported by survey participants (Table 3). Average daily PCP use was greater among female participants (41.0%) than among male participants (24.6%). Exceptions included facial moisturiser (male participants: 59%, female participants: 22%; *p* < 0.001), fragranced shampoo (male participants: 52%, female participants: 40%; *p* = 0.01) and fragranced shaving cream (male participants: 16%, female participants: 18%; *p* = 0.5).

**Table 3.** Daily use frequency of PCPs by sex reported by surveyed participants from 2013 and between 2016 and 2017^a^

PCP preparation category	Individual PCPs	Female n = 496 (%)	Male n = 248 (%)	*p*-value
Eye make-up	Eyeliner (liquid or pencil)	43	7	**<0.001**
	Brow pencil	24	3	**<0.001**
	Eye shadow	24	2	**<0.001**
	**Average eye make-up use**	**30.3**	**4.0**	
**Other make-up**	Make-up primer	19	2	**<0.001**
	Liquid foundation or concealer	39	2	**<0.001**
	Powder foundation or concealer	33	2	**<0.001**
	Lip balm, lipstick, or lip gloss	77	27	**<0.001**
	Blush or bronzing make-up	42	5	**<0.001**
	Lip pencil	11	3	**<0.001**
	Make-up remover	40	2	**<0.001**
	**Average other make-up use**	**37.3**	**21.5**	
All make-up	**Average make-up use**	**35.2**	**5.5**	
Manicuring	**Nail polish^b^**	**8**	**2**	**<0.001**
Body care: skin care	Facial moisturiser	22	59	**<0.001**
	Hand or body lotion	67	41	**<0.001**
	Sunscreen	29	8	**<0.001**
	**Average skin care product use**	**39.3**	**36**	
Hair	**Hairstyling products**	**34**	**31**	**<0.001**
Other body care	Deodorant or antiperspirant	**84**	**76**	0.05
Fragrance	Fragranced shampoo	40	52	**0.01**
	Fragranced conditioner	40	31	**<0.001**
	Fragranced soap or body wash	66	64	0.4
	Fragranced facial soap or cleanser	55	37	**<0.001**
	Fragranced shaving cream	16	18	0.5
	Perfume or cologne or body spray	56	30	**<0.001**
	Fragranced hand soap	75	61	**0.001**
	**Average fragranced product use**	**48.9**	**41.9**	
	**Average PCP use by sex**	**41.0**	**24.6**	

^a^Participants who responded either “more than once a day” or “daily” use were grouped together to comprise daily use.

^b^Comparing “never” use to all other categories.

Bold numbers indicate the average use per personal care product preparation category. Bold and italic numbers indicate statistical significance.

### Associations of use frequency of individual PCPs and racial/ethnic category

There were significant associations between the frequency of use of some PCPs and racial/ethnic categories, some of these associations persisted in the adjusted models (Table 4). One such association was seen with NHB participants who reported more frequent use of hand lotion [proportionate odds ratios (pOR): 4.16; 95% CI confidence interval (CI): 2.71–6.43], hair products (pOR: 3.29; 95% CI: 2.16–4.99) and lip gloss/lip balm (pOR: 2.75; CI: 1.76–4.36), but less frequent use of shampoo (pOR: 0.26; CI: 0.17–0.39), conditioner (POR: 0.49; CI: 0.33–0.73) and sunscreen (pOR: 0.43; CI: 0.26–0.69) when compared to NHW participants (Table 4).

**Table 4.** Associations (OR and CIs) of race/ethnic category with frequency use of PCPs (n = 753)

PCP	Race/ethnicity												
	NHW (n = 264)	Non-hispanic black (n = 107)		Asian (n = 150)		Hispanic (n = 94)		MENA (n = 48)		Multiracial (n = 34)		Race unspecified (n = 56)	
		Unadjusted	Adjusted^a^	Unadjusted	Adjusted^a^	Unadjusted	Adjusted^a^	Unadjusted	Adjusted^a^	Unadjusted	Adjusted^a^	Unadjusted	Adjusted^a^
Face lotion	1.00 (Ref)	1.32 (0.88-1.97)	***1.59 (1.05-2.44)***	***1.79 (1.24-2.59)***	***2.02 (1.36-3.04)***	1.14 (0.75-1.74)	1.19 (0.76-1.85)	1.64 (0.96-2.84)	1.22 (0.69-2.16)	1.24 (0.66-2.36)	1.59 (0.83-3.08)	1.40 (0.82-2.39)	1.67 (0.97-2.91)
Hand lotion	Ref	***3.28 (2.18-4.97)***	***4.16 (2.71-6.43)***	***1.84 (1.28-2.66)***	***2.03 (1.36-3.04)***	***1.74 (1.15-2.64)***	***1.79 (1.16-2.79)***	***2.16 (1.24-3.79)***	1.65 (0.93-2.95)	1.54 (0.82-2.93)	1.82 (0.96-3.47)	***1.73 (1.03-2.94)***	***2.05 (1.19-3.53)***
Hair products	Ref	***3.08 (2.06-4.64)***	***3.29 (2.16-4.99)***	0.96 (0.66-1.39)	0.93 (0.62-1.39)	1.19 (0.77-1.81)	1.17 (0.76-1.81)	1.59 (0.94-2.71)	1.33 (0.77-2.29)	1.02 (0.52-1.95)	1.09 (0.56-2.11)	1.71 (0.99-2.92)	1.71 (0.99-2.95)
Sunscreen	Ref	***0.41 (0.25-0.66)***	***0.43 (0.26-0.69)***	0.96 (0.65-1.40)	0.91 (0.59-1.38)	0.92 (0.59-1.44)	0.86 (0.54-1.36)	1.13 (0.61-2.04)	0.77 (0.41-1.42)	0.59 (0.28-1.19)	0.69 (0.32-1.41)	0.62 (0.35-1.08)	0.59 (0.32-1.05)
Deodorant	Ref	1.36 (0.89-2.09)	1.47 (0.95-2.29)	***0.42 (0.28-0.62)***	**0.49 (0.32-0.76)**	1.14 (0.73-1.78)	1.30 (0.82-2.07)	0.79 (0.45-1.41)	0.87 (0.48-1.57)	0.64 (0.33-1.24)	0.64 (0.33-1.25)	0.73 (0.42-1.28)	0.83 (0.47-1.47)
Shampoo	Ref	***0.28 (0.18-0.41)***	***0.26 (0.17-0.39)***	0.92 (0.64-1.31)	0.87 (0.59-1.29)	0.86 (0.56-1.32)	0.84 (0.54-1.31)	0.71 (0.41-1.23)	0.85 (0.48-1.49)	0.94 (0.50-1.76)	0.84 (0.45-1.59)	0.79 (0.46-1.35)	0.76 (0.44-1.31)
Conditioner	Ref	***0.48 (0.33-0.71)***	***0.49 (0.33-0.73)***	1.04 (0.73-1.49)	1.11 (0.75-1.64)	1.21 (0.79-1.86)	1.24 (0.80-1.92)	0.83 (0.49-1.43)	0.84 (0.48-1.46)	1.33 (0.71-2.49)	1.31 (0.69-2.49)	0.95 (0.57-1.59)	1.03 (0.61-1.75)
Body soap	Ref	**1.83 (1.19-2.82)**	***1.84 (1.20-2.85)***	1.07 (0.75-1.54)	1.09 (0.74-1.62)	1.29 (0.84-1.99)	1.32 (0.85-2.06)	***1.87 (1.03-3.43)***	***2.02 (1.09-3.75)***	1.29 (0.69-2.46)	1.27 (0.67-2.44)	0.96 (0.56-1.68)	0.99 (0.57-1.75)
Face soap	Ref	**1.81 (1.20-2.73)**	***1.93 (1.27-2.94)***	***2.05 (1.42-2.95)***	***2.01 (1.36-2.99)***	***2.21 (1.45-3.39)***	***2.21 (1.45-3.39)***	***1.77 (1.01-3.09)***	1.74 (0.98-3.07)	1.82 (0.95-3.47)	1.85 (0.96-3.52)	1.64 (0.95-2.81)	1.66 (0.96-2.89)
Shaving cream	Ref	**1.58 (1.06-2.37)**	1.49 (0.99-2.24)	1.35 (0.92-1.97)	1.13 (0.75-1.69)	***1.85 (1.20-2.84)***	***1.68 (1.08-2.61)***	1.06 (0.59-1.89)	1.02 (0.55-1.85)	1.67 (0.84-3.29)	1.56 (0.78-3.09)	1.33 (0.77-2.25)	1.17 (0.67-1.99)
Perfume	Ref	**2.49 (1.66-3.76)**	**2.57 (1.69-3.91)**	1.41 (0.98-2.04)	1.17 (0.79-1.75)	***2.36(1.56-3.59)***	***2.04 (1.32-3.16)***	***4.05 (2.35-7.01)***	***3.33 (1.89-5.89)***	1.79 (0.95-3.42)	1.88 (0.98-3.59)	1.28 (0.76-2.15)	1.20 (0.70-2.04)
Hand soap	Ref	1.09 (0.71-1.68)	1.18 (0.76-1.83)	0.73 (0.51-1.05)	0.76 (0.51-1.13)	1.02 (0.66-1.56)	1.07 (0.69-1.67)	***2.21 (1.21-4.19)***	***2.16 (1.16-4.19)***	***2.17 (1.07-4.62)***	***2.20 (1.08-4.73)***	0.59 (0.35-1.03)	0.64 (0.37-1.12)
Lip gloss, lip balm, lipstick	Ref	**1.93 (1.27-2.95)**	**2.75 (1.76-4.36)**	1.21 (0.84-1.75)	1.39 (0.93-2.11)	1.03 (0.68-1.56)	1.03 (0.66-1.61)	1.55 (0.87-2.81)	1.18 (0.64-2.19)	1.59 (0.85-3.06)	***2.21 (1.13-4.46)***	0.90 (0.54-1.52)	1.12 (0.65-1.93)
Blush	Ref	1.17 (0.77-1.77)	1.36 (0.86-2.13)	1.19 (0.81-1.75)	1.28 (0.83-1.97)	1.27 (0.81-1.97)	1.22 (0.75-1.95)	***2.65 (1.51-4.66)***	***2.03 (1.12-3.68)***	1.13 (0.56-2.23)	1.37 (0.65-2.76)	0.88 (0.49-1.54)	1.07 (0.57-1.95)
Eye liner	Ref	0.91 (0.59-1.38)	1.05 (0.66-1.65)	1.12 (0.76-1.64)	1.46 (0.94-2.25)	1.08 (0.69-1.68)	1.12 (0.68-1.83)	***1.81 (1.05-3.11)***	1.23 (0.69-2.21)	0.99 (0.50-1.89)	1.24 (0.60-2.49)	1.09 (0.64-1.85)	1.37 (0.75-2.45)
Eye shadow	Ref	***0.61 (0.38-0.97)***	0.73 (0.44-1.20)	***0.65 (0.43-0.97)***	0.76 (0.48-1.21)	0.73 (0.45-1.16)	0.71 (0.42-1.17)	0.86 (0.47-1.54)	0.64 (0.34-1.19)	0.64 (0.29-1.31)	0.76 (0.34-1.62)	***0.54 (0.28-0.98)***	0.69 (0.35-1.32)
Liquid foundation	Ref	***0.60 (0.38-0.92)***	0.66 (0.40-1.08)	0.83 (0.56-1.22)	1.02 (0.64-1.62)	0.75 (0.47-1.18)	0.73 (0.44-1.21)	1.27 (0.71-2.26)	0.91 (0.48-1.70)	0.87 (0.42-1.74)	1.14 (0.53-2.42)	0.63 (0.35-1.09)	0.79 (0.42-1.46)
Powder foundation	Ref	0.73 (0.46-1.14)	0.82 (0.50-1.34)	0.89 (0.59-1.32)	1.09 (0.69-1.74)	0.89 (0.56-1.41)	0.91 (0.55-1.51)	1.13 (0.61-2.03)	0.87 (0.46-1.64)	0.71 (0.32-1.45)	0.85 (0.37-1.83)	0.61 (0.33-1.11)	0.79 (0.41-1.52)
Brow pencil	Ref	***1.97 (1.21-3.17)***	***2.52 (1.51-4.22)***	1.55 (0.98-2.45)	***1.93 (1.16-3.23)***	1.52 (0.89-2.55)	1.69 (0.95-2.94)	***2.99 (1.63-5.44)***	***2.55 (1.34-4.80)***	1.83 (0.85-3.77)	***2.35 (1.05-5.03*)**	1.19 (0.59-2.28)	1.60 (0.77-3.22)
Lip pencil	Ref	***1.79 (1.00-3.15)***	***2.09 (1.15-3.79)***	1.42 (0.81-2.45)	1.48 (0.81-2.69)	1.24 (0.63-2.35)	1.25 (0.62-2.69)	***3.61 (1.83-6.98)***	**2.87 (1.41-5.71)**	1.36 (0.49-3.31)	1.62 (0.57-4.04)	1.47 (0.68-3.01)	1.64 (0.73-3.49)
Nail polish	Ref	1.21 (0.77-1.90)	1.37 (0.83-2.22)	0.83 (0.54-1.28)	0.97 (0.59-1.60)	1.49 (0.93-2.37)	1.59 (0.96-2.62)	1.01 (0.52-1.89)	0.88 (0.44-1.75)	1.33 (0.64-2.63)	1.55 (0.72-3.21)	0.62 (0.31-1.17)	0.79 (0.38-1.58)
Primer	Ref	0.92 (0.53-1.57)	1.12 (0.63-1.95)	1.29 (0.81-2.05)	***1.74 (1.03-2.92)***	1.43 (0.84-2.39)	1.56 (0.88-2.72)	***2.20 (1.18-4.02)***	***2.00 (1.04-3.80)***	1.28 (0.54-2.77)	1.59 (0.66-3.58)	0.83 (0.39-1.64)	1.14 (0.52-2.33)
Make-up remover	Ref	0.86 (0.55-1.32)	1.03 (0.63-1.65)	1.24 (0.84-1.83)	***1.71 (1.08-2.69)***	1.32 (0.83-2.09)	1.59 (0.95-2.64)	**1.86 (1.05-3.28)**	1.64 (0.88-3.00)	0.74 (0.34-1.53)	0.93 (0.40-2.06)	0.76 (0.42-1.34)	1.06 (0.55-1.99)

^a^Adjusted for country of birth, level of education, age and sex.

Ref = the reference group of non-Hispanic white (NHW) participants.

### Risk perception of PCPs

Most NHW participants agreed that PCPs were safe, while those who identified as non-White were more likely to disagree (Table 5). There were no statistically significant differences by race or ethnicity for regulation and protection of PCPs. However, more Asian (54%) and NHW (53%) participants agreed PCPs were sufficiently regulated compared to Hispanic (37%) and MENA (44%) participants. NHW participants were more likely to agree that PCPs were safe (79%) while the proportion of NHB participants to agree was significantly lower (51%) (*p* = 0.03). This pattern in responses was also observed when participants were asked if chemical additives are safer now than in the past (NHW: 60%; NHB: 32%; *p* = 0.01). MENA participants were more likely to believe there are health risks associated with PCPs (74%) compared to NHW (54%) (*p* = 0.04). At least 80% of respondents in each race/ethnic group agreed that the government should be responsible for ensuring ingredient safety in PCPs.

**Table 5.** Participant responses to risk perception statements on PCPs by race and ethnicity (n = 768)

Risk perception statement	Agree	MENA n = 50 n (%)	Asian or Asian American n = 154 n (%)	Black or African American n = 109 n (%)	Hispanic or Latino n = 96 n (%)	Multiracial n = 35 n (%)	NHW or Caucasian n = 267 n (%)	Other n = 57 n (%)	*p*-value
Regulations for chemicals in commerce protect consumers	Y	14 (33)	53 (42)	36 (40)	24 (33)	16 (52)	83 (44)	20 (49)	0.75
	N	20 (48)	46 (37)	37 (41)	36 (49)	12 (39)	73 (39)	14 (34)	
PCPs are sufficiently regulated	Y	21 (44)	82 (54)	51 (47)	35 (37)	16 (47)	141 (53)	27 (47)	0.41
	N	18 (38)	39 (25)	38 (35)	37 (39)	10 (29)	71 (27)	18 (32)	
Government protects consumers and immediately reports health risks associated with ingredients in PCPs	Y	18 (38)	77 (51)	44 (41)	31 (33)	11 (32)	109 (41)	20 (36)	0.09
	N	27 (56)	51 (34)	49 (45)	50 (53)	16 (47)	128 (48)	26 (46)	
Chemical industry protects consumers and immediately reports health risks associated with ingredients in PCPs	Y	13 (27)	61 (40)	39 (36)	37 (39)	9 (26)	92 (34)	19 (34)	0.47
	N	28 (58)	65 (43)	58 (54)	43 (45)	17 (50)	141 (53)	31 (55)	
Health risks are associated with use of PCPs	Y	37 (74)	100 (65)	76 (70)	64 (67)	23 (66)	143 (54)	39 (68)	***0.04***
	N	9 (18)	30 (20)	22 (20)	19 (20)	8 (23)	88 (33)	11 (19)	
PCPs are safe	Y	25 (51)	116 (75)	76 (70)	67 (71)	25 (74)	211 (79)	38 (67)	***0.03***
	N	16 (33)	23 (15)	21 (19)	18 (19)	5 (15)	43 (16)	11 (19)	
If PCPs contained a harmful ingredient, I would not purchase it	Y	41 (85)	131 (87)	88 (81)	76 (80)	32 (94)	228 (86)	50 (89)	0.55
	N	7 (15)	14 (9)	17 (16)	16 (17)	1 (3)	29 (11)	5 (9)	
Chemical additives are safer today than they were in the past	Y	15 (32)	79 (53)	60 (56)	47 (49)	17 (50)	161 (60)	27 (48)	***0.01***
	N	25 (53)	42 (28)	32 (30)	27 (28)	13 (38)	60 (22)	22 (39)	
Manufacturers should be responsible for ensuring the ingredients in PCPs are safe for consumers	Y	47 (94)	138 (90)	91 (84)	88 (93)	34 (100)	249 (94)	51 (89)	0.18
	N	1 (2)	9 (6)	12 (11)	4 (4)	0 (0)	10 (4)	4 (7)	
Government should be responsible for ensuring the ingredients in PCPs are safe for consumers	Y	46 (94)	127 (84)	89 (82)	85 (89)	32 (94)	218 (82)	48 (86)	**0.01**
	N	1 (2)	13 (9)	14 (13)	6 (6)	2 (6)	44 (16)	6 (11)	
Independent organisations should be responsible for ensuring the ingredients in PCPs are safe for consumers	Y	42 (86)	126 (83)	87 (81)	81 (85)	31 (91)	221 (83)	48 (86)	0.50
	N	4 (8)	13 (9)	17 (16)	8 (8)	2 (6)	35 (13)	5 (9)	
I would trust the chemical and/or cosmetic industry to provide reliable information regarding the safety of PCPs	Y	18 (38)	73 (49)	42 (39)	47 (49)	15 (44)	117 (44)	19 (35)	0.21
	N	27 (56)	60 (40)	58 (54)	45 (47)	18 (53)	134 (50)	31 (56)	
I would trust the government to provide reliable information regarding the safety of PCPs	Y	21 (44)	89 (60)	57 (53)	52 (55)	17 (50)	138 (52)	31 (55)	0.81
	N	21 (44)	49 (33)	42 (39)	36 (38)	16 (47)	110 (41)	21 (38)	
I would trust scientists to provide reliable information regarding the safety of PCPs	Y	38 (79)	114 (77)	72 (68)	77 (81)	29 (85)	230 (86)	50 (89)	***0.01***
	N	7 (15)	23 (15)	29 (27)	15 (16)	4 (12)	29 (11)	6 (11)	
I would trust a consumer association to provide reliable information regarding the safety of PCPs	Y	26 (54)	99 (66)	60 (56)	56 (59)	24 (71)	180 (67)	35 (62)	0.28
	N	16 (33)	38 (25)	41 (38)	30 (32)	9 (26)	65 (24)	19 (34)	
I would trust media outlets to provide reliable information regarding the safety of PCPs	Y	10 (21)	51 (34)	37 (34)	24 (25)	7 (21)	62 (23)	17 (30)	***0.003***
	N	33 (69)	77 (51)	63 (58)	63 (66)	27 (79)	190 (71)	35 (62)	
The specific components of ‘fragrance’ in PCPs should be listed as ingredients	Y	40 (80)	113 (73)	81 (75)	75 (79)	27 (79)	200 (75)	43 (75)	0.46
	N	3 (6)	18 (12)	18 (17)	7 (7)	3 (9)	26 (10)	9 (16)	
PCPs should be required to list all ingredients present in the product	Y	45 (94)	135 (89)	93 (87)	91 (96)	34 (100)	254 (95)	52 (95)	*0.01*
	N	1 (2)	8 (5)	11 (10)	1 (1)	0 (0)	7 (3)	3 (5)	

Bold and italic numbers indicate statistical significance.

Key: Y = yes; N = no.

More Asian participants reported they would trust the government to provide reliable information on PCP safety than any other group (60%) and while at least 65% of respondents from each group indicated they would trust scientists for this information, NHW participants (86%) were more likely to do so than NHB participants (68%) (*p* = 0.01). There were high levels of distrust in media outlets to provide reliable information across racial and ethnic groups; however, the multiracial participants had the highest level of distrust of media (79%) compared to Asian respondents with the lowest level (51%) (*p* = 0.003).

Most participants across groups indicated that they agreed the chemicals found in fragrances should be specifically listed and PCPs should be required to list all ingredients in the products ranging from 87% agreement in the Black participant group to 100% agreement in the multiracial group (Table 5).

## Discussion

In the present study, we evaluated PCP use trends and assessed risk perception associated with PCPs among a racially/ethnically diverse sample of adults sampled from a US university institution and online. We found that PCP use patterns and risk perception on PCP use varied by race/ethnicity with the highest daily use on average and the perception that health risks are associated with use of PCPs reported more frequently by MENA participants compared to other participants.

Moreover, in general, perception of risks associated with use of PCPs differed between the racial/ethnic groups as we observed differences in the consensus on who should be responsible for ensuring product safety as well as communicating this to the public. However, over 80% of participants in each racial/ethnic group agreed that PCPs should be required to list all ingredients present in the product and that the government should be responsible for ensuring product safety. In terms of our population of college attending students, our results aligned with similar studies where female college student participants had a higher use frequency of PCPs [30]. Our results of risk perceptions in our MENA participants were similar to those of a paper with Saudia Arabian female students [31] where, while participants were aware of chemicals in cosmetic products, they used the products at least once daily.

Recently, the federal Modernization of Cosmetics Regulation Act of 2022 (MoCRA) was passed, which will be paramount in increasing government accountability in ensuring PCP safety. Provisions that are outlined in this legislation include the requirement adverse event reporting to the FDA within 15 business days, the FDA should be provided access to review records when requested, product manufacturers and processors should register their facilities with the FDA as well as report updated lists of product ingredients in PCPs to the FDA annually [32].

Overall, participants reported trusting scientists to provide reliable information on PCP safety, but this level of trust was less prevalent when government and industry were considered. This also indicates the vital role that scientists have in informing and educating the public in increasing environmental health awareness. Moreover, interdisciplinary partnerships, including academia, community organisations and health communication experts, are needed to determine the best approach to develop and disseminate this information to the general public through social media platforms and traditional media avenues.

Our findings of high use frequency as well as higher levels of risk perception among this population may also be indicative of an environmental health awareness issue and lack of knowledge on current regulations that are not protective, lack of product transparency and/or knowledge of resources that guide consumers on PCP products. Thus, the passing of the MoCRA in late 2022 is significant and speaks to the reality that while participants trust scientists with the provision of reliable PCP information, there was a much-needed framework through which product transparency should be reported to the FDA and the consumer.

While over half of the participants in each racial/ethnic group agreed they would trust scientists to provide reliable information regarding the safety of PCPs, the lowest frequency was reported among NHB participants (68%). We posit that this may be attributed to the history of unethical and inhumane experimentation in the US, which typically used Black persons as the research subjects. The knowledge of these experiments in addition to current experiences of discrimination and racism in medicine and science has contributed to the established mistrust of science and research from Black people. This illustrates the critical role of inclusive and participatory research with communities of colour and other marginalised communities. Researchers must be intentional, transparent and willing to work towards building trust with the communities they wish to include in their research. This process, while typically slow-going, is worthwhile to develop genuine and sustained community–academic partnerships that have positive impacts, both within the research domain, but also for the populations involved [33–42].

NHW participants were significantly more likely to agree that PCPs are safe, that chemical additives are safer today than they were in the past, and to believe that there are no health risks associated with the use of PCPs compared to non-White participants. This is consistent with prior studies that indicate non-White racial/ethnic groups do not feel they have the proper knowledge about chemicals in PCPs [10,20,43]. This perception may be due to past lived experiences of racism in addition to medical and environmental injustices, as women of colour and low-income individuals are known to more frequently be exposed to social stressors and environmental hazards [13,44–48], have fewer choices available to them in terms of product quality and are frequent victims of unethical medical and environmental practices.

Knowing the history of discriminatory practices against their racial and ethnic group creates distrust [34,39,49–52] and is an explanation for non-White participants’ perception that current chemical regulations do not offer adequate protection. Nonetheless, despite this distrust, there is significant daily use of PCPs within the groups in this sample population. Future research is warranted to examine why participants continue to use PCPs even with significant perception of the potential risks involved. This is especially important as persistent exposures to EDCs in these PCPs can pose a significant potential health disparities risk.

PCP use occurred more frequently among MENA participants for individual PCPs, particularly brow pencil, lip pencil, blush and make-up remover, compared with other racial and ethnic groups. While more than half of the MENA participants reported more frequent use of PCPs than participants who identified as another race or ethnicity, this racial/ethnic group disagreed that the chemical industry actively works to protect consumers and will immediately report any health risks associated with the ingredients in PCPs. It is plausible that there may be some cognitive dissonance in recognising potential risks in using PCPs [53]. This contrasts with external societal influences, which are stronger factors when maintaining cultural and social beauty standards [13,54,55].

While this is the first study of its kind to compare PCP use between MENA participants and those of other racial and ethnic groups, previous studies have outlined how societal pressures force, mainly, women of colour to conform to Eurocentric beauty standards [56–58], such as possessing lighter skin complexion versus dark [56]. The desire to be perceived as conventionally attractive has been the reason for the frequent use of select PCPs and subsequent differential exposures to EDCs within these products [10,55,59].

The use of lip products was more frequent among multiracial participants and, in accordance with previous studies, NHB participants were significantly more likely to use hand or body lotion and hairstyling products on a daily basis [10,14,60–63]. While MENA participants reported high levels of sunscreen use, NHB were more likely not to use sunscreen on a daily basis. Culturally, the use of sunscreen and other measures of protection from sun exposure is widespread in Arabic countries and populations [64–66]. It should be highlighted that sunscreen formulations contain ultraviolet (UV) filters which are also known to contain EDCs [67]. Previous studies have outlined that Arabic women particularly sought to avoid sun exposure and were averse to having their skin tanned or darkened [64]. Conversely, there is an enduring misconception that due to elevated levels in melanin found in the skin of darker persons, there is increased protection from the effects of UV ray harm and thus sunscreen use is not a frequent practice within Black or African American populations [68,69].

Similar to the 2021 study by Collins et al. conducted among 70 NHB, 73 Latina, 78 Vietnamese, 79 NHW and 18 mixed race women [10], the use of shampoo and conditioner was less frequently reported by Black participants. While in their study Hispanic/Latino participants had the highest frequency of make-up use, MENA participants in our study were more likely to use make-up preparations compared to all other racial/ethnic groups [10]. NHW participants were more likely to report daily use of fragranced shampoos and conditioners compared to other participants. Those who identified as Hispanic or Latino were the least likely overall to report daily use of all make-up preparations on average as well as nail polish use. In the 2021 Collins paper, their NHW participants had the highest household income while Latina and Vietnamese participants had the lowest [10] with other data revealing that the 2021 median household income for Hispanic households in the US was approximately $58,000 while that of NHW households was an estimated $78,000 [70]. Thus, while this study did not directly capture the socioeconomic status (SES) of participants, these racial/ethnic differences in PCP product use may be due to socioeconomic disparities, particularly in terms of household and disposable income. Make-up and nail polish are more cosmetic items rather than personal hygiene items, they are also products that are not necessarily needed for everyday activities, and it is cost-effective to use them less frequently if someone’s economic situation does not allow for spending on non-essential items.

This study had some limitations. Participants were recruited from a college campus; thus, the participants are likely not generalisable to the general population by age and education level. In terms of representativeness of our data, a major strength of our study was the diversity of participants. Our study population was fairly young, and so may not be representative of older and less diverse populations. Nearly all participants had completed high school or some college or were college graduates. Thus, our findings may not represent those with less than high school education. In addition, one third of participants were born outside the US, therefore our study may be representative of more international populations. Additionally, while the sample data are not exactly representative of the general US population, the major racial/ethnic groups are included in this work. We acknowledge future research that includes more participants from minoritised populations is needed. Another limitation of this work was that we did not ask if participants were aware of the history of racial discrimination and its impact on their perceptions of safety and trust in the products. Capturing this information would be important for future studies as racial and ethnic minoritised groups and low-income individuals are more frequently exposed to social stressors and environmental hazards [4] and at earlier ages. Additional limitations were the lack of inclusion of specific hair products (e.g., hair relaxers, texturising, salon services) as well as other PCPs, such as menstrual hygiene products, which are recognised sources of endocrine-disrupting chemicals [71,72]. Finally, in this study, we did not capture or measure the SES of the respondents. However, while we did not measure SES in our study, other studies have shown that there is an association between SES and the purchasing and use of PCPs ([42,73]). Specifically, this relationship may depend on the SES and spending power of the individual. Future studies should incorporate these measures.

Despite the limitations noted, this study had several strengths. The study was large in terms of sample size and the first to evaluate use trends and perceptions of risk with PCP use across a diverse group of racial and ethnic groups with our study including MENA and multiracial groups, which has not been done previously. There was minimal missingness by race, with only a small percentage (between 1-4%) not being included in analysis (Appendix D). We also evaluated several types of PCPs including make-up, body care and fragranced products in this study. Additionally, the online nature of the survey provided increased access for participation by respondents who may have been excluded from the study through in-person recruitment alone.

## Conclusions

In summary, consistent with prior research, this study found that both PCP use and perceptions of risk in PCP use varied by race and ethnicity. Further research in other settings is needed to determine if these differences in use and risk perception between racial and ethnic groups are consistent to inform public health intervention and environmental policies. There were significant differences in daily use frequency, levels of trust, perception of safety and health risks associated with PCPs by race and ethnicity among young adults.

## Data Availability

The datasets generated during and/or analysed during the current study are available from the corresponding author on reasonable request.

## Appendix

**Appendix A.** List of personal care products (PCPs) that participants were asked about in terms of use frequency

List of personal care items
Lip balm, lipstick or lip gloss Blush or bronzing make-up Eyeliner (liquid or pencil) Eye shadow Liquid foundation or concealer Powder foundation or concealer Brow pencil Lip pencil Nail polish Make-up primer Make-up remover Facial moisturiser Hand or body lotion Sunscreen Deodorant or antiperspirant Hairstyling products Fragranced shampoo Fragranced conditioner Fragranced soap or body wash Fragranced facial soap or cleanser Fragranced shaving cream Perfume or cologne or body spray Fragranced hand soap

**Appendix B.** List of risk perception statements that were posed to survey participants

Risk perception statement category	No.	Risk perception statement
Regulation and protection	1.	Regulations for chemicals currently in commerce are adequate to protect consumers.
	2.	PCPs are sufficiently regulated.
	3.	The government actively works to protect consumers and will immediately report any health risks associated with the ingredients in PCPs.
	4.	The chemical industry actively works to protect consumers and will immediately report any health risks associated with the ingredients in PCPs.
Risk and safety	5.	There are health risks associated with the use of PCPs.
	6.	PCPs are safe.
	7.	If a PCP contained an ingredient I knew to be harmful, I would not purchase it.
	8.	Chemical additives are safer today than they were in the past.
Responsibility	9.	Manufacturers should be responsible for ensuring the ingredients in PCPs are safe for consumers.
	10.	The government should be responsible for ensuring the ingredients in PCPs are safe for consumers.
	11.	Independent organisations should be responsible for ensuring the ingredients in PCPs are safe for consumers.
Trust	12.	I would trust the chemical and/or cosmetic industry to provide reliable information regarding the safety of PCPs.
	13.	I would trust the government to provide reliable information regarding the safety of PCPs.
	14.	I would trust scientists to provide reliable information regarding the safety of PCPs.
	15.	I would trust a consumer association to provide reliable information regarding the safety of PCPs.
	16.	I would trust media outlets to provide reliable information regarding the safety of PCPs.
Transparency	17.	The specific components of ‘fragrance’ in personal care products should be listed as ingredients.
	18.	PCPs should be required to list all ingredients present in the product.

**Appendix C.** Daily use frequency of PCPs by country of birth reported by surveyed participants from 2013 and between 2016 and 2017^a^

PCP preparation category	Individual PCP	US		Non-US		*p*-value
		n = 515	%	n = 236	%	
Eye make-up	Eyeliner (liquid or pencil)	161	**3**	73	**31**	0.2
	Brow pencil	87	**17**	41	**17**	0.07
	Eye shadow	93	**18**	31	**13**	0.2
	**Average**		**12.7**		**20.3**	
Other make-up	Make-up primer	71	**14**	26	**11**	0.5
	Liquid foundation or concealer	141	**27**	57	**24**	0.2
	Powder foundation or concealer	122	**24**	50	**21**	0.4
	Lip balm, lipstick, or lip gloss	313	**61**	141	**60**	0.8
	Blush or bronzing make-up	153	**30**	70	**30**	0.1
	Lip pencil	41	**8**	23	**10**	0.6
	Make-up remover	142	**28**	59	**25**	0.4
	**Average**		**27.4**		**25.9**	
All make-up	**Average make-up use**		**23**		**24.2**	
Manicuring	**Nail polish^b^**	28	**5**	17	**7**	0.3
Body care: skin care	Facial moisturiser	183	**36**	77	**33**	0.3
	Hand or body lotion	289	**56**	148	**63**	0.3
	Sunscreen	101	**20**	65	**28**	0.08
	**Average skin care product use**		**37.3**		**41.3**	
Hair	**Hairstyling products**	164	**32**	84	**36**	0.8
Other body care	**Deodorant or antiperspirant**	446	**87**	167	**71**	**<0.001**
Fragrance	Fragranced shampoo	230	**45**	102	**43**	0.8
	Fragranced conditioner	200	**39**	78	**33**	0.3
	Fragranced soap or body wash	351	**68**	141	**60**	**0.01**
	Fragranced facial soap or cleanser	254	**49**	115	**49**	**0.002**
	Fragranced shaving cream	75	**15**	47	**20**	0.05
	Perfume or cologne or body spray	225	**44**	128	**54**	**0.01**
	Fragranced hand soap	373	**72**	155	**66**	**0.04**
	**Average fragranced product use**		**47.4**		**46.4**	

^a^Participants who responded either “more than once a day” or “daily” use were grouped together to comprise daily use.

^b^Comparing “never” use to all other categories.

Bold numbers indicate the average use per personal care product preparation category. Bold and italic numbers indicate statistical significance.

**Appendix D.** Table showing missingness of data for regression analysis

Personal information	Table 1	Number missing in regression analyses	% Missing
Ethnicity			
Middle Eastern and North African	50	48	4
Asian or Asian American	154	150	2.6
Black or African American	109	107	1.8
Hispanic or Latino	96	94	2.1
Multiracial	35	34	2.9
Non-Hispanic White or Caucasian	267	264	1.1
Other	57	56	1.8
Total	**768**	**753**	**1.9**
Gender			
Female	502	496	1.2
Male	258	248	3.9
Non-binary/Prefer not to answer	9	0	100
Total	**769**	**744**	**3.3**
Country of Birth			
US	525	515	1.9
Outside of the US	242	236	2.5
Total	**767**	**751**	**2.1**
