# Correction to: Risk perception and use of personal care products by race and ethnicity among a diverse population

**DOI:** 10.14324/111.444/ucloe.3038.CORR

**Published:** 2024-06-26

**Authors:** Julia Mandeville, Zeina Alkhalaf, Charlotte Joannidis, Michelle Ryan, Devon Nelson, Lesliam Quiros-Alcala, Matthew O. Gribble, Anna Z. Pollack

**Affiliations:** 1 Department of Global and Community Health, College of Public Health, George Mason University, Fairfax, VA, USA; 2 Department of Environmental Health and Engineering, Johns Hopkins University, Baltimore, MD, USA; 3 Division of Occupational, Environmental & Climate Medicine, University of California, San Francisco, CA, USA

The original article can be found online at: Mandeville J, Alkhalaf Z, Joannidis C, Ryan M, Nelson D, Quiros-Alcala L, Gribble MO, Pollack AZ. Risk perception and use of personal care products by race and ethnicity among a diverse population. *UCL Open: Environment*. 2024;(6):04. Available from: https://doi.org/10.14324/111.444/ucloe.3038

The correct Table 1 is provided (data for Other, under Ethnicity/Race is now in the correct line).

**Table 1.** Socio-demographic characteristics of survey participants (n = 770)^a^

Personal information	Mean ± SD
Age (years)	22.82 ± 6.03
	n (%)
Ethnicity/Race	n = 768
MENA	50 (6.5)
Asian or Asian American	154 (20.1)
Black or African American	109 (14.2)
Hispanic or Latino	96 (12.5)
Multiracial	35 (4.6)
NHW or Caucasian	267 (34.8)
Other	57(7.4)
Gender	n = 769
Female	502 (65.3)
Male	258 (33.5)
Nonbinary/Prefer not to answer	9 (1.2)
Country of birth	n = 767
US	525 (68.4)
Outside of US	242 (31.6)
Education	n = 769
High school/some college	626 (81.4)
College graduate	117 (15.2)
Other	26 (3.4)

^a^Participants were enrolled in two phases: 2013 and 2016–2017.

